# Stall in fertility decline in Eastern African countries: regional analysis of patterns, determinants and implications

**DOI:** 10.1098/rstb.2009.0166

**Published:** 2009-10-27

**Authors:** Alex C. Ezeh, Blessing U. Mberu, Jacques O. Emina

**Affiliations:** African Population and Health Research Center, Shelter Afrique Centre, Longonot Road, Upper Hill, PO Box 10787-00100, Nairobi, Kenya

**Keywords:** fertility stall, sub-Saharan Africa, fertility transition

## Abstract

We use data from the Demographic and Health Surveys to examine the patterns of stall in fertility decline in four Eastern African countries. Contrary to patterns of fertility transition in Africa that cut across various socio-economic and geographical groups within countries, we find strong selectivity of fertility stall across different groups and regions in all four countries. In both Kenya and Tanzania where fertility decline has stalled at the national level, it continued to decline among the most educated women and in some regions. While fertility has remained at pre-transition level in Uganda over the past 20 years, there are signs of decline with specific groups of women (especially the most educated, urban and those in the Eastern region) taking the lead. For Zimbabwe, although fertility has continued to decline at the national level, stall is observed among women with less than secondary education and those in some of the regions. We link these intra-country variations to differential changes in socio-economic variables, family planning programme environment and reproductive behaviour models. The results suggest that declines in contraceptive use, increases in unmet need for family planning, increasing preferences for larger families, and increases in adolescent fertility were consistently associated with stalls in subgroup fertility across all four countries. These results are consistent with models that emphasize the role of declines in national and international commitments to family planning programmes in the premature stall in sub-Saharan fertility transition.

## 1. Introduction

Slowing population growth has been identified as a necessary, though not sufficient, factor in ameliorating foreseeable threats to global peace and prosperity. The persistent high fertility in sub-Saharan Africa (SSA) and its potential adverse effects on the region's development efforts led to substantial research, policy and action focused on identifying and addressing the various social, cultural and economic factors that served to maintain fertility at high levels in the region (Shapiro & Gebreselassie 2008; Bongaarts & Sinding 2009). Apart from efforts to increase educational opportunities and improve health conditions, the main policy response to concerns about rapid population growth was the implementation of voluntary family planning programmes that provided information about, and access to contraceptives that permitted women and men to control their reproductive lives and reduce unwanted childbearing (Bongaarts & Sinding 2009). By the beginning of the 1990s, a corpus of studies documented evidence of the spread of fertility decline in some countries throughout the developing world, including SSA. Total fertility rates (TFR) were projected to decline in both Asia (from 4.2 to 2.4) and Latin America and the Caribbean (from 3.5 to 2.5) between 1985 and 2005. Although the projected decline for SSA was not as sharp as in the other regions, TFR in the region was projected to decline from 6.5 to 5.2 over the same period (US Census Bureau 1998).

In the recent past, however, new concerns have emerged that the ongoing fertility decline in Africa has been stalling since the second part of the 1990s and early 2000s in some countries that have been at the forefront of fertility decline in the region. An analysis of fertility trends in countries with multiple nationally representative datasets identified about 15 African countries as experiencing stall^1^ in fertility decline (Bongaarts 2006, 2008; Westoff & Cross 2006; Garenne 2007; Moultrie *et al*. 2008; Schoumaker 2008; Shapiro & Gebreselassie 2008). Several countries in the region, however, are still at an early stage of fertility transition (e.g. Tanzania, Rwanda, Mozambique and Guinea), while some others are at pre-transition fertility levels (e.g. Mali, Niger and Uganda; Shapiro & Gebreselassie 2008).

With slow pace of fertility decline in many SSA countries, growing evidence of stall at high fertility levels of more than five children per woman in a third of the countries, and several other countries remaining at pre-transition or early stages of fertility transition, fertility decline in Africa is thought to have considerably slowed in the second part of the 1990s and early 2000s (Bongaarts 2008). The potential implications of these trends for the region's social and economic development have engendered new research interests, debates and discourses, including the role of voluntary family planning as a principal policy instrument in addressing high rates of population growth in the region (Bongaarts & Sinding 2009).

Although the stalling of fertility decline in some African countries has been evidently documented, there are little consensus on the magnitude and causes of such stalls. Published studies have offered several hypotheses and explanations for the stall in fertility decline but with mixed results. The factors that have been linked to the stall include the loss of focus on family planning programmes in the development agenda of these countries following new health challenges such as HIV/AIDS (Agyei-Mensah 2007); the HIV/AIDS epidemic and its impact on infant and child mortality (Westoff & Cross 2006; Moultrie *et al*. 2008); changes in proximate determinants of fertility including changing attitudes towards family size preferences (Bongaarts 2002, 2006, 2008; Westoff & Cross 2006); changes in attitudes towards family planning; changes in levels of contraceptive use and socio-economic development (as reflected in changes in women's education, infant and child mortality and real *per capita* economic growth (Bongaarts 2006; Westoff & Cross 2006; Shapiro & Gebreselassie 2008).

In their detailed study of Kenya, Westoff & Cross (2006) illustrated the selectivity of stall in fertility decline, showing that the stall is particularly selective of subgroups with certain socio-economic characteristics. For instance, while the stall in Kenya is identifiable among women with lower or no formal education, fertility decline, though marginal, continued among the most educated women. Likewise, stall in contraceptive prevalence is seen mainly among younger women and among those with less education. These previous findings raise several relevant research and policy questions:

- does the selectivity of stall in fertility decline observed in Kenya apply to other countries in the region?
- what are the drivers of the stall in fertility decline and do these drivers vary across subgroups? and
- what are the implications of the stall on the future population of the SSA region?

In seeking answers to these questions, we reiterate what is known about stalled fertility decline in SSA, underscoring issues relating to patterns and drivers of the stall. We quantify the magnitude of the fertility change, highlighting specificities and commonalities across four^2^ countries in Eastern Africa (Kenya, Tanzania, Uganda and Zimbabwe) and within subregions and subgroups in each country. In particular, we examine why specific subgroups within a country experience an increase or stagnation in their fertility behaviour while other subgroups within the same country continue to experience a decline in their fertility. We draw implications of our findings for the future of the region's population size and policy directions.

## 2. Objectives

Following from our stated research questions, this study specifically seeks to address the following objectives:

- describe fertility trends, patterns and magnitude of stalled fertility decline in four Eastern African countries at the national levels and among subgroups within the countries;
- identify unique and common factors associated with stalling or continuing fertility decline among subgroups within each country;
- highlight the implications of the patterns, magnitude and drivers of the stall for the future population size of the region; and
- identify possible policy and programmatic strategies for addressing fertility stall in the region.

## 3. Analytical framework

Most of the literature seeking to explain fertility patterns in SSA can be classified into one of three models in terms of the factors they hold as important in explaining observed levels, differentials or trends in fertility. These are the *reproductive behaviour model, the socio-economic model and the institutional model*. Changes in fertility, including both fertility transition and stall in the transition, have also been explained by changes associated with these models. Changes in fertility may result from changes in the reproductive preferences or behaviour of individual women; changes in the compositional distribution of women by different socio-economic characteristics that are associated with fertility; or changes in the institutional policy or programme environment that may constrain childbearing decisions of individual women or limit their access to reproductive health services. The effects of changes in the institutional policy or programme environment may operate through changes in individual preferences or behaviour. We summarize below the main propositions of each of these models with special focus on stalled fertility transition, how we operationalize them in the current study, and their respective importance in understanding stall in fertility decline in the Eastern Africa region. Again, we analyse these changes at the regional level within each country.

### (a) Change in reproductive behaviour model

According to the reproductive behaviour model, changes in proximate determinants of fertility could explain changes in fertility, including *fertility stalls*. The model primarily focuses on changes in fertility preferences or behaviour. In general, declining or steady age at marriage, earlier start of motherhood, shorter birth intervals, increasing out-of-wedlock childbearing, decreasing contraceptive use and/or declining infertility rates could slow or stall fertility decline (Bongaarts 2006, 2008; Garenne 2007; Shapiro & Gebreselassie 2008). For instance, the average annual rate of increase in contraceptive prevalence among countries with stalling fertility was 0.8 per cent per year compared to 1.4 per cent per year observed among the non-stalling mid-transitional countries (Bongaarts 2006). Likewise, fertility stall in Kenya and Rwanda was associated with either a decline or no improvement in contraceptive use, while the tempo of marriage and childbearing has been noted as factors explaining stalling fertility in Tanzania (Garenne 2007).

Following empirical and theoretical considerations and the availability of data across the four countries in this study, we focus on changes in contraceptive use, changes in desired family size, and changes in adolescent childbearing as the key variables of interest in our examination of the role of changes in reproductive behaviour on the fertility stall in Eastern Africa. Although we do not have data for all countries and all surveys on attitudes towards family planning, we also examine the role of changes in attitude towards family planning by women of childbearing age and their spouses, in the countries and periods for which the data exist, in explaining stall in fertility transition in Eastern Africa. Initial evidence of declines in fertility rates in most SSA countries suggested that the declines were preceded or followed by significant increases in contraceptive prevalence and decreases in desired family size. In the present study, rather than simply establish if the stall varies across subgroups, we further demonstrate how these proximate fertility determinants drive observed trends in fertility within and across countries. These reproductive behaviour factors may be influenced by several other factors including the level of support for family planning programmes in the country. While adolescent fertility (per cent of adolescents aged 15–19 who have become mothers) is directly related to fertility outcomes, it also captures both the changing tempo and changing onset of childbearing which are known to influence overall fertility rates (Emina 2005; Garenne 2008).

### (b) Institutional model: family planning service environment

One of the conclusions that has received far more consensus among scholars of sub-Saharan fertility transition is the role played by organized family planning programmes in initiating fertility decline in the region. Growing interest by many developing country governments to adopt national population policies between the late 1960s and mid 1990s was matched by phenomenal growth in international funding assistance for family planning programmes. Although only two developing country governments had official policies to support family planning programmes in 1960, this number had increased to 74 by 1975 and 115 by 1996 (Cleland 2009). Between 1971 and 1985, international funding for family planning programmes grew from about $168 million to $512 million annually (United Nations Population Fund, UNFPA 1988). By 1990, fertility decline was well underway across Asia and Latin America and had started in SSA. The institutional model recognizes the strength of this force (Cutright & Kelly 1981; Davanzo & Adamson 1998), and with respect to stall in fertility transition, the institutional model argues that the loss of international and/or national focus on family planning programmes in SSA since the mid-1990s is a major factor in the stall. The 10-year review of the flow of financial resources for the implementation of the programme of action of the International Conference on Population and Development^3^ reported that donor support for family planning commodities and service delivery fell from US $560 million to US $460 million between 1995 and 2003. Other estimates covering longer periods show how the proportion of donor assistance for family planning dwindled relative to other competing new challenges such as HIV/AIDS since the mid 1990s. For instance, Van Dalen & Reuser (2008) show that between 1995 and 2007, the share of family planning in international development assistance declined from about 54 per cent to less than 5 per cent in 2006 and 2007. During the same period, the proportion of international assistance to sexually transmitted diseases/HIV/AIDS increased from under 10 per cent to over 85 per cent.

Scholars adopting the institutional model have specifically linked the stall in fertility decline to shifts in the observed level of support for family planning programmes either at the local, national or international levels. Under this perspective, changing patterns of unmet need for family planning, increasing levels of unwanted childbearing, declining knowledge of family planning methods or sources, increasing negative attitudes towards family planning, decreasing importance of public institutions as a key source of family planning services, etc., have all been pointed to as consequences of this shift in priority and hence, as drivers of the stall in fertility decline. Other related measures of the strength of this hypothesis include the proportion of national budgets allocated to family planning services, the proportion of international development assistance going into family planning programmes, number of organizations and personnel working in the area of family planning, and the amount of media coverage for family planning programmes (Davanzo *et al*. 1998). Unfortunately, the dearth of data in operationalizing and measuring these factors at country and regional levels has limited the full exploration of the impact of these factors in the stall in fertility transition in SSA. In our analyses, we focus on changes in unwanted fertility rates and role of the public sector in the provision of family planning services. The latter is measured by changes in the proportion of modern contraceptive users who obtained their last method from a public facility. We argue that, given the largely uniform participation of various subgroups in the initial fertility decline, a differentiated stall in fertility decline may be related to differentiated access to family planning services in the face of diminished supply or excess demand for services. We further argue that women who led in the stall may come from underserved subgroups and will therefore be expected to have higher levels of unwanted childbearing and lower contraceptive use.

### (c) Socio-economic model

Perhaps the most dominant theme in the explanation of fertility levels, differentials and trends is the socioeconomic model. Both within and across countries, differentials and changing fertility patterns have been explained largely as resulting from socio-economic and sometimes socio-cultural differences among groups. At the risk of oversimplification, the socio-economic approach to fertility analyses largely assumes that high fertility is an economically rational response (Caldwell 1982; Lipton 1983; Stecklov 1999). There are costs and benefits of having children (e.g. educational costs or benefit from children's work and old-age security) and these benefits outweigh the costs in high fertility settings. Fertility changes as the relative valuation of these costs and benefits change, and there are key socio-economic characteristics that drive these changes in the relative valuation of costs and benefits of having children. In particular, women's education, female labour force participation, urban residence, household wealth, cultural norms (often measured by religion or ethnicity), and overall levels of social development (often measured by region or level of urbanization) have been central to explanations of fertility levels and differentials. While these factors may affect fertility through changes in their compositional distributions in a population (Ezeh & Dodoo 2001; Bongaarts 2002, 2006, 2008; Schoumaker 2004; Garenne 2007; Shapiro & Gebreselassie 2008), what is of interest largely is their behavioural effect on fertility. For example, earlier work on fertility transition in the region has particularly emphasized the importance of women's education in contributing to fertility decline, both directly and via the proximate determinants of marriage and contraceptive use, as well as via the influence it has on infant and child mortality (Shapiro & Gebreselassie 2008).

Although available evidence suggests that groups defined by several of these factors participated in the initial fertility decline in SSA, they have varied in their experiences of the stall in the fertility decline. We re-evaluate the contribution of these socio-economic factors to initial declines in fertility and, focusing on each characteristic, we assess how specific behavioural and institutional shifts have affected the relative contribution of each characteristic to the stall in fertility decline. We argue that shifts in family planning programme environment may affect different subgroups of women differently. This will lead to different behavioural outcomes and hence, differential contributions to stall in fertility decline. In our analyses, we focus on female education, place of residence and household living standard. Further, we examine changes in the proportion of women in professional employment in each region. We examine differentials and trends in fertility rates by each of these characteristics and then assess how the various institutional and behavioural factors explain the observed trends in each region.

**Figure 1.**
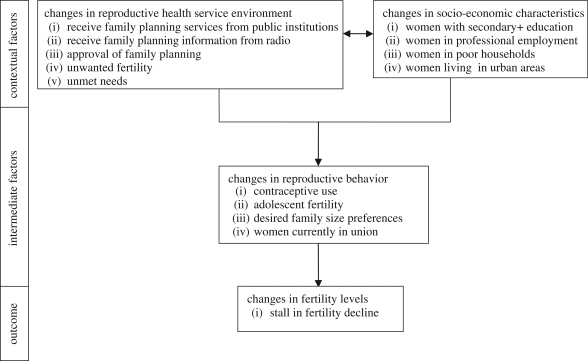
Analytical framework for the determinants of stalled fertility decline.

Following from the framework discussed above and availability of data, we summarize the analytical framework for this study in figure 1. The framework assumes that changes in institutional environment relating to the provision of family planning information and services (measured in terms of proportion of female contraceptive users who received family planning services from public facilities, proportion of women who received family planning information through the mass media, direction of changes in attitudes towards family planning and levels of unwanted fertility), and changes in socio-economic characteristics (measured by the proportion of women with secondary or higher education, proportion of women in professional employment, proportion of women in poor households and proportion of urban residents) constitute the contextual factors that influence reproductive preferences and behaviour, including stall in fertility decline. We measure reproductive behaviour by four key variables: changes in the level of contraceptive use, adolescent fertility, family size preferences and proportion of women in union.

## 4. Data and methods

### (a) Data

We use secondary data from the Demographic and Health Surveys (DHS) programme for four East African countries that have had four rounds of national surveys as described in table 1. The focus on countries with four rounds of national surveys is necessary to establish both decline in fertility rates and stall in observed fertility decline. The dates of the surveys vary, with the latest survey conducted in 2007 and the earliest in 1988. The years between the first and last surveys for each country are summarized in column 2 of table 1.

**Table 1.** Countries included in the study and years of respective surveys.

country	year of survey (DHS)	years covered by all surveys	no. of regions
Kenya	1989, 1993, 1998, 2003	14	7
Tanzania	1992, 1996, 1999, 2004	12	6
Uganda	1988, 1995, 2000/2001, 2007	19	4
Zimbabwe	1988, 1994, 1999, 2005/2006	18	10

Overall, data from 16 surveys were analysed for 27 regions of the four countries. TFR and other reproductive behaviour indicators (contraceptive use, adolescent fertility, etc.) were calculated for each survey by the defined socio-economic, demographic and reproductive health service environment variables and by region. Changes in each indicator were defined by comparing estimates between two consecutive surveys. Whereas the majority of previous studies have focused on national level analyses, we based our analysis at the regional level. This approach allows us, following Westoff & Cross (2006), to identify intra-country/spatial homogeneity or heterogeneity in the stall in fertility decline.

Generally, female education, urban residence and household wealth have been identified with initial adoption of contraceptive use and small family size values in SSA. These factors have also been linked to explanations of the stall in fertility decline in the region (Uchudi 2001; Bongaarts 2002; Garenne & Joseph 2002; Shapiro & Tambashe 2002; Shapiro *et al*. 2003). Shapiro & Gebreselassie (2008) in particular have noted the importance of the direct and indirect effects of these factors (like female education) in influencing fertility outcomes. Our analysis re-examines the role of these and other variables in explaining changing fertility patterns at the regional level in four Eastern African countries.

### (b) Measures

*Predictors*: *changes in reproductive health service environment, socio-economic development and reproductive behaviour factors are our three key predictors of stalled fertility decline*.

We use change in the proportion of women who got their last contraceptive method from a public facility, change in the proportion of women who received family planning information through the mass media, and change in unwanted TFRs as proxies for changes in the family planning service environment. Other variables considered under this model include changes in attitudes towards family planning (measured by changes in the proportion of women and men who approve of family planning) and changes in proportion of women with unmet need for family planning. As national and international support for family planning programmes waned, the public sector may become less important as the main provider of family planning services; family planning information, education and communication services may suffer leading to less favourable dispositions towards family planning; and unwanted childbearing may increase as demand for services outstrip supply. Although, some of these variables are missing in some of the survey years, especially for the most recent period for family planning attitude variables, we examine their bivariate relationships with stall in fertility decline for the countries where they are available.

We use changes in proportion of the most educated women (secondary level or higher), changes in proportion of females living in urban areas, changes in proportion of women in professional employment, and change in proportion of women living in poor households^4^ to capture changes in socio-economic and demographic characteristics of women that influence fertility outcomes. Both the family planning service environment and the socio-economic characteristics operate through a set of proximate factors to influence fertility outcomes.

#### (i) Intermediate factors

For changes in reproductive behaviour, we examined changes in contraceptive prevalence, adolescent fertility, family size preferences and proportion of women in union. Changes in prevalence of modern contraceptive use and ideal number of children measure changes in the motivation for fertility control, while change in adolescent fertility captures the change in the tempo of reproductive behaviour beyond age at first marriage and age at first birth, as unmarried childbearing continues to assume increasing significance in the region (Emina 2005; Garenne 2008). We include change in the proportion of women in union in our model as control for exposure to pregnancy risk.

#### (ii) Outcome

Stall in fertility decline is our main outcome variable. ‘Stall’ is a dummy variable created by comparing fertility levels in the three most recent surveys. If change during the third period is equal to or greater than zero (meaning that TFR in the most recent survey is equal to or higher than TFR in the third survey) while change in the previous two periods is negative (meaning that TFR has been declining in the prior surveys), ‘stall’ equals 1. ‘Stall’ equals 0 in all other cases. This definition of stall is consistent with that used by previous studies that defined stall as an interruption period in an ongoing fertility transition before a country reaches the end of the transition (Gendell 1985; Bongaarts 2006; Moultrie *et al*. 2008).

Given differences in the duration of the period between two consecutive surveys within and across countries, we normalized the change to reflect a five year period which is more common across the surveys. We divide the observed difference by the duration between the two surveys and multiply the result by five.

### (c) Methods of analysis

To identify the factors associated with stalled fertility decline, we use descriptive and multivariate methods. We examine the relationships between observed changes in institutional, socio-economic and reproductive behaviour variables and change in TFR across the four countries and all the regions within each country. Descriptive method includes analysis of change in fertility for the last inter-survey period compared with change in each explanatory variable for the same period. For the multivariate analysis, we employ a probit regression model.

#### (i) The probit model

Given that our main outcome is a dummy variable (stall or no stall) and predictors are continuous, we implemented probit models whose likelihood function follows the binomial distribution. We estimated the probit model using a maximum likelihood function in the form:
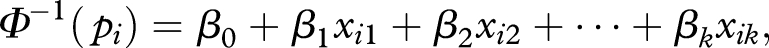
where (i) *p*_*i*_ is the probability that *y_i_* = 1 and *Φ*^−1^(*p*_i_) is the inverse of the cumulative distribution function of a standard normal variable. As probability ranges between 0 and 1, the probit function ranges between −∞ and +∞; and (ii) *x*_*i*1_, … ,*x*_*ik*_ are independent variables.

The interpretation of a probit coefficient, *b*, is that a one-unit increase in the predictor leads to an increase in the probit score by *b* standard deviations.

#### (ii) Analysis strategy

The analysis is performed in three stages. First, we assess the crude effect of each independent variable on the outcome (bivariate analysis). Second, we performed analysis by the theoretical model presented in the analytical framework (institutional, socio-economic and reproductive behaviour). We ran a separate model for each set of explanatory variables to identify the best predictors among the selected independent variables. We examined the independent variables for multicollinearity, and in cases where two or more independent variables were highly correlated, we only use one of the variables. Lastly, we included all significant variables from each model in the final model to capture their net effect.

## 5. Results

### (a) National fertility trends

Table 2 presents overall trends in fertility at the national level in each of the four countries. It shows both the annual rate of change in fertility rates over the period covered by the four surveys in each country and a normalized change over the most recent inter-survey period.

**Table 2.** TFR trends by countries.

country	survey				TFR (%) change	
	DHS1^a^	DHS2^a^	DHS3^a^	DHS4^a^	annual	third period (5 years)
Kenya	6.7	5.4	4.7	4.9	1.9	4.3 (stall)
Tanzania	6.2	5.8	5.6	5.7	0.7	1.8 (stall)
Uganda	7.4	6.9	6.9	6.7	0.5	−5.2 (decrease)
Zimbabwe	5.4	4.3	4.0	3.8	1.65	−6.0 (decrease)

^a^Dates of each survey are reported in table 1.

Two distinct fertility trends are evident across the four countries. Uganda and Tanzania which, by the most recent survey period, still had TFRs of 6.7 and 5.7 children per woman, respectively, experienced the least annual per cent change in TFR at 0.5 and 0.7 per cent, respectively, while Kenya and Zimbabwe experienced annual rates of decline that were 2.5 to 3.8 times larger.

Focusing on the most recent inter-survey period, however, Kenya and Tanzania show clear reversals in their fertility decline with TFR in their latest survey edging higher than in the penultimate survey. Uganda and Zimbabwe continued to experience a decline in TFR, albeit at different fertility levels. For Zimbabwe, the rate of decline has continued to decrease over the past 15 years, while in the case of Uganda, fertility only slightly declined in most recent periods, but changed very little over the 19-year period covered by the surveys. In fact, TFR remains at pre-transitional level at 6.7 children per woman by 2007 in Uganda.

These four countries indeed represent three fertility regimes: Uganda represents a country at pre-transitional fertility level but one that is poised to enter the phase of incipient demographic transition; Kenya and Tanzania started their fertility transition much earlier but are currently experiencing clear stall in their fertility decline; and Zimbabwe represents a country where fertility decline, though ebbing, has continued to progress.

### (b) Dynamics of fertility trends within countries

Regional analysis of fertility trends shows different patterns within the same country (table 3). Of the 27 regions in the four countries, only in one region (Eastern region in Uganda) has fertility remained unchanged at 7.5 children per woman across the 19 years covered by the surveys. Indeed, in three of the four regions in Uganda, TFR ranged between 6.8 and 7.6 children per woman in 2007. In 60 per cent of the regions, fertility continued to decline in the most recent inter-survey period, although very marginally in some. In 10 of the 27 regions, stall in fertility decline was evident, where fertility levels in the most recent survey was equal to or higher than the fertility level in the third survey and the region had previously recorded a measurable decline in fertility. Although Kenya and Tanzania experienced a stall at the national level, the regional patterns are quite different. In Kenya, all regions, except Central and Coast, experienced a stall in fertility decline while in Tanzania, only two regions experienced a stall in fertility decline. In Uganda and Zimbabwe, where fertility declined at the national level, most regions recorded declining fertility patterns, yet in three regions of Zimbabwe, fertility stall was evident. Zimbabwe demonstrates the wide regional variations in fertility trends being experienced in SSA. Although all regions experienced substantial declines in fertility between 1988 and 1994 in Zimbabwe, by the most recent period, two regions, Bulawayo and Masvingo, differed significantly in their fertility patterns with fertility declining in Bulawayo by 23 per cent between 1998 and 2003 while Masvingo experienced a 26 per cent increase in fertility during the same period. These results show that regions within the same country may not only experience huge differentials in fertility levels but trends could be in opposite directions.

**Table 3.** Trends in total fertility rates at regional level in Eastern Africa, DHS 1988–2007. DHS statcompiler (measuredhs.com).

region	TFR1	TFR2	TFR3	TFR4	change (%)	comment
Kenya	1989	1993	1998	2003	TFR 3 → 4	
Nairobi	4.20	3.40	2.60	2.70	3.85	stall
Central	6.00	3.90	3.70	3.40	−8.11	declining
Coastal	5.40	5.30	5.00	4.90	−2.00	declining
Eastern	7.20	5.90	4.70	5.10	8.51	stall
Nyanza	6.90	5.80	5.00	5.60	12.00	stall
Rift valley	7.00	5.70	5.30	5.80	9.43	stall
Western	8.10	6.40	5.60	5.80	3.57	stall
total	6.70	5.40	4.70	4.90	4.26	stall
Tanzania	1992	1996	1999	2004	TFR 3 → 4	
Coastal	5.70	4.90	4.30	4.00	−6.98	declining
Northern Highlands	6.00	5.70	5.10	4.90	−3.92	declining
Lake	6.90	7.00	7.10	7.00	−1.41	declining
Central	7.10	6.10	5.40	6.10	12.96	stall
Southern Highlands	6.30	5.40	5.20	5.90	13.46	stall
South	5.10	4.90	5.00	4.80	−4.00	declining
total	6.20	5.80	5.60	5.70	1.79	stall
Uganda	1988	1995	2000	2006/2007	TFR 3 → 4	
Central	6.90	6.30	5.70	5.20	−8.77	declining
Eastern	7.50	7.40	7.40	7.60	2.70	increasing
Northern	7.40	6.80	7.90	7.40	−6.33	declining
Western	7.80	7.00	6.90	6.80	−1.45	declining
total	7.40	6.90	6.90	6.70	−2.90	declining
Zimbabwe	1988	1994	1999	2006	TFR 3 → 4	
Manicaland	5.90	4.50	4.70	4.20	−10.64	declining
Mashonaland Central	5.20	4.60	4.90	4.60	−6.12	declining
Mashonaland East	5.80	4.80	4.20	3.70	−11.90	declining
Mashonaland West	5.70	4.80	4.10	3.70	−9.76	declining
Matabeleland North	6.60	5.80	4.10	4.20	2.44	stall
Matabeleland South	5.50	5.00	4.80	4.00	−16.67	declining
Midlands	5.80	4.50	4.00	4.20	5.00	stall
Masvingo	6.40	4.60	3.90	4.90	25.64	stall
Harare/Chitungwiza	3.90	2.80	3.00	2.50	−16.67	declining
Bulawayo	3.50	3.20	3.00	2.30	−23.33	declining
total	5.40	4.30	4.00	3.80	−5.00	declining

This pattern of wide regional variation in fertility trends within the same country is evident across the four Eastern African countries. In Kenya, Central region experienced an 8 per cent decline in fertility between 1998 and 2003 while Nyanza, Rift valley and Eastern provinces experienced increases in fertility rates of between 9 and 12 per cent. In Tanzania, fertility declined in Coastal region by 7 per cent while Southern Highlands and Central regions experienced increases in fertility rates of 14 and 13 per cent, respectively. Irrespective of fertility trends at the national level, each country contains regions that are experiencing continued declines in fertility as well as those experiencing stalls in their fertility decline. Regional analysis of fertility trends may therefore provide better understanding and explanations of stalls in fertility declines than analysis at the national level. Understanding what changes in family planning service environment, socio-economic and demographic characteristics and behavioural factors that drive these regional patterns is essential to explaining and addressing the stall in fertility decline in SSA. In the bivariate analysis, we focus attention on the regions within each country that have widely contrasting fertility patterns to assess changes in the contextual and proximate fertility determinants between the third and fourth DHS surveys.

The differences in fertility patterns observed across regions within the same country may also exist among other subgroups defined by socio-economic and other characteristics. In table 4, we present experience of fertility stall in the most recent inter-survey period by education and place of residence. Consistent with earlier studies showing that fertility declined across all socio-economic groups, we note that all educational groups and urban and rural women experienced a decline in fertility between the first- and second-DHS surveys in each of the four countries, although the change was marginal in some groups. During the most recent inter-survey period, these groups differed substantially in their fertility patterns within countries. Table 4 highlights substantial variations in fertility trends in Kenya, Tanzania, Uganda and Zimbabwe by female education and place of residence. In Kenya and Tanzania, for instance, despite national stalls in fertility decline, fertility continued to decline among the most educated women. By contrast, however, in Uganda which experienced fertility decline nationally, we observe a stall among the most educated and urban women. Likewise, the national fertility decline in Zimbabwe contrasts with stalled decline among women with no education. The effects of these variables, therefore, cannot be assumed to be uniform across countries or regions within countries. Understanding their roles in stalled fertility decline within specific contexts will be essential to fully understanding the drivers of the stall in fertility decline in SSA.

**Table 4.** Total fertility rates in Kenya, Tanzania, Uganda and Zimbabwe by female education and place of residence. DHS statcompiler (measuredhs.com).

	DHS 1	DHS 2	DHS 3	DHS 4	per cent change period 3→4	fertility trend
Kenya	1989	1993	1998	2003		
*educational level*						
no education	7.5	6	5.8	6.7	15.52	stall
primary	6.9	5.7	5	5.5	10	stall
secondary or higher	4.9	4	3.5	3.2	−8.57	declining
*place of residence*						
urban	4.5	3.4	3.1	3.3	6.45	stall
rural	7.1	5.8	5.2	5.4	3.85	stall
total	6.7	5.4	4.7	4.9	4.26	stall
Tanzania	1992	1996	1999	2004		
*educational level*						
no education	6.5	6.4	6.5	6.9	6.15	stall
primary	6.3	5.6	5.2	5.6	7.69	stall
secondary or higher	4.2	3.2	3.5	3.3	−5.71	declining
*place of residence*						
urban	5.1	4.1	3.2	3.6	12.50	stall
rural	6.6	6.3	6.5	6.5	0	stall
total	6.2	5.8	5.6	5.7	1.79	stall
Uganda	1988	1995	2000/2001	2006/2007		
*educational level*						
no education	7.9	7	7.8	7.7	−1.28	declining
primary	7.2	7.1	7.3	7.2	−1.37	declining
secondary or higher	5.6	5.2	3.9	4.4	12.82	stall
*place of residence*						
urban	5.7	5	4	4.4	10	stall
rural	7.6	7.2	7.4	7.1	−4.05	declining
total	7.4	6.9	6.9	6.7	−2.90	declining
Zimbabwe	1988	1994	1999	2005/2006		
*educational level*						
no education	7.2	5.2	5.2	5.8	11.54	stall
primary	5.7	4.6	4.5	4.5	0	stall
secondary or higher	3.7	3.3	3.3	3.2	−3.03	declining
*place of residence*						
urban	3.8	3.1	3	2.6	−13.33	declining
rural	6.2	4.9	4.6	4.6	0	stall
total	5.4	4.3	4	3.8	−5	declining

The selectivity of fertility change observed across different regions, socio-economic groups and urban and rural areas within and across countries support findings from previous studies in the region (Westoff & Cross 2006; Garenne 2008). For instance, Garenne (2008) noted that even though all SSA countries appear to be undergoing a fertility decline; the fertility dynamics and their impact are often different in different regions and among specific subgroups.

These findings raise one key question: what factors are driving regional and other subgroup differentials in fertility trends within and across countries? To answer this question, we first compare changes in institutional and socio-economic factors associated with fertility trends in SSA as well as changes in proximate determinants of fertility as defined in our analytical framework with changes in TFR at the regional level. Through this process, we identify both unique and common factors associated with stall (or continuing fertility decline) in each region across all four countries. We further identify the common factors that determine overall fertility change at regional level across all four countries in our study by implementing multivariate regression models to assess the net effect of each of the factors defined in our framework. The results of the relationships between change in TFR and the institutional, socio-economic and reproductive behaviour factors in all regions of the four countries discussed in the subsequent sections are summarized in tables S1–S3, respectively in the electronic supplementary material.

### (c) Unique factors associated with stalling fertility decline at regional level

#### (i) Changes in family planning service environment

Tables S1–S3 in the electronic supplementary material provide trends in each of the variables included in our explanatory models. We use logarithmic scale to compute change in each of the variables within our three explanatory models of stalled fertility decline and present these in tables 5–7 below. This is to account for the wide variation in absolute change in most of the variables given differences in levels at the penultimate survey. We show in these tables the ratio of change between the last survey and the preceding survey. We also show fertility pattern at each region between the last two surveys as discussed in table 3. Nationally, we observe an increase or no change in unwanted fertility rates across all countries—with the exception of Zimbabwe—where unwanted fertility declined by about 10 per cent. Use of public facilities as source of family planning declined in Kenya and Zimbabwe by about 8 per cent—while it increased in Tanzania and Uganda by 2 and 6 per cent, respectively. Information on approval of family planning was not available in Uganda and Zimbabwe for the most recent survey. In Kenya, approval of family planning declined by 10 per cent while it increased by 12 per cent in Tanzania. There was no change in unmet need levels in all countries except Uganda where a 13 per cent increase was observed. Information on family planning on radio declined in Uganda and Zimbabwe while it increased marginally in Tanzania. In summary, fertility stall in Kenya is associated with a significant increase in unwanted fertility, decrease in the role of public sector in the provision of family planning and decreases in the positive attitude towards family planning. None of these factors are significantly associated with the stall in Tanzania or the marginal decline in Uganda. Indeed, the only significant family planning service environment factor at the national level is an increase in positive attitude towards family planning in Tanzania and an increase in unmet need and decrease in family planning information on radio in Uganda. Zimbabwe, which clearly continues to experience a decline in fertility, witnessed a significant decrease in unwanted fertility despite declines in the role of public sector in the provision of family planning services and the promotion of family planning information on radio.

How do these national patterns differ across regions with divergent trends in fertility? Again, as table 3 shows, in Kenya, Central region experienced a continued decline in fertility while Nyanza, Rift Valley and Eastern regions experienced substantial increases in TFR between the 1998 and 2003 surveys. Central region is the only region in Kenya where unwanted fertility declined significantly by 61 per cent between the two most recent DHS surveys, although the region also experienced a significant decline in the role of public facilities in the provision of family planning services and a marginal decline in approval of family planning. In Nyanza region, unwanted fertility increased substantially by 33 per cent—while the role of public facilities and approval of family planning declined significantly by 14 and 7 per cent, respectively. Consequently, unmet need in the region increased substantially by 27 per cent. The pattern in the other regions with substantial increases in fertility is similar to Nyanza region. Indeed, almost all the increases in negative attitude towards family planning occurred in the region with substantial increases in fertility.

In Tanzania, Coastal region experienced the largest decline in TFR between 1999 and 2004. In this region, unwanted fertility and unmet need for family planning declined significantly by 22 and 32 per cent, respectively. Approval of family planning and access to family planning information on the radio also increased significantly by 7 and 3 per cent, respectively. In the two regions that drove the increase in fertility in Tanzania (Central and Southern Highlands), unwanted fertility increased substantially by 59–61%. However, while unmet need for family planning significantly increased in Central region, it declined significantly in Southern Highlands. In Zimbabwe, the role of the public sector in the provision of family planning services and access to family planning information on the radio declined across all provinces, suggesting an overall deterioration in the family planning service environment. The only exception is Matabeleland North where the role of the public sector increased significantly by 6 per cent. However, while unmet need for family planning increased significantly in Matabeleland North, it decreased in Midlands. These results suggest that changes in the family planning service environment may be a significant determinant of stall in fertility transition in Eastern Africa.

### (d) Changes in socio-economic factors

We use four variables to assess the impact of changes in socio-economic factors on the stall in fertility decline in Eastern Africa: change in the per cent of women living in urban areas, change in per cent with secondary or higher education, change in the proportion working in professional jobs and change in the proportion living in poor households. Across all four countries, percentage living in urban areas increased significantly in Kenya and Tanzania. Percentage of women with secondary or higher education increased in all countries except Kenya while the percentage working in professional jobs decline significantly in Kenya and Zimbabwe, while it increased in Tanzania and Uganda. The percentage of women living in poor households declined significantly in all countries except Zimbabwe although this may not reflect improved economic status as the variables used to define poverty, especially in urban areas may reflect more the differential availability of certain services and amenities in some geographical locations.

Part of the difficulty of modelling the impact of changes in these explanatory variables on observed fertility patterns at the regional level is that regions vary enormously on these indicators, with some approximating the maximum observable value while others are at very low levels. For instance, while the percentage of urban has been 100 per cent in Nairobi region across all surveys (meaning change is zero), the percentage of urban in Eastern region was less than 1 per cent in 1988, increasing to 9 per cent by 2003. Again, focusing on regions with divergent fertility trends, we assess the extent to which observed changes in these socio-economic factors are related to observed changes in fertility trends in each region.

In Kenya, Central region experienced substantial increases in percentage of women living in urban areas, proportion with secondary or higher education, and percentage working in professional jobs. Indeed, it is the only region where the proportion of women working in professional jobs actually increased given a significant decrease of 19 per cent at the national level. In addition, the proportion of women living in poor households in Central region declined by 68 per cent between 1998 and 2003. Nyanza and Eastern regions experienced significant declines in the proportion living in urban areas while Eastern and Western regions experienced significant declines in the proportion of women with secondary or higher education.

In Tanzania, Coastal region which experienced the largest decline in fertility recorded significant increases in the proportion of women living in urban areas and in those with secondary or higher education between 1999 and 2004, as well as an increase in the proportion working in professional jobs. These factors declined in Southern Highlands which experienced the highest increases in fertility during the same period. In Uganda, Eastern region where fertility remained high with signs of increasing trends, the proportion of women living in urban areas significantly declined between 2000 and 2007. In Zimbabwe, the proportion of women with secondary or higher education increased significantly at the national level between 1999 and 2006. This increase was also significant in almost all regions except those experiencing a stall in their fertility decline. The proportion of Zimbabwean women in professional jobs declined significantly at the national level but this decline is only significant in two of the three regions experiencing a stall in fertility decline.

### (e) Changes in reproductive behaviour factors

As noted earlier, changes in socio-economic and family planning service environment may influence fertility stall through its influence on the reproductive behaviour of individual women. Limited programmatic emphasis on family planning could reverse gains in promoting small family size values and use of contraception. Adolescents may be disproportionately affected as available services are biased towards women in union. We examine, at both the national and regional levels, how changes in these reproductive behaviour factors are related to observed changes in fertility trends. Overall, there were no significant changes in desired family size across countries and regions. Contraceptive prevalence increased significantly in Tanzania and Zimbabwe but did not change in Kenya and Uganda. While adolescent fertility increased in Kenya, it declined significantly in Uganda and only marginally in Tanzania and Zimbabwe. The proportion of women in union declined significantly in all countries except Tanzania where a marginal increase was observed.

Again, we assess how these national trends operate at regional levels in each country given observed differentials in fertility trends at the regional level. Central region of Kenya is the only region in Kenya that experienced a significant decline in the proportion of women in union. Contraceptive prevalence increased in the region and in Western region while it declined in all the other regions. Nyanza province, which recorded the highest increase in fertility, also experienced a significant decline in contraceptive prevalence at 17 per cent. The fertility increase in Nairobi appears to have been driven largely by increases in adolescent childbearing, where the proportion of 15–19 year olds who have become mothers increased by 50 per cent.

In Tanzania, Southern Highlands region, which recorded the highest increases in fertility between 1999 and 2004, is the only region that experienced a decline in contraceptive prevalence. Adolescent childbearing also increased significantly in Southern Highlands region by 43 per cent while Coastal region which recorded the highest decline in fertility experienced a significant decline in adolescent childbearing at 36 per cent. In Zimbabwe, although contraceptive prevalence increased significantly at the national level and across all regions, marginal declines were observed in two of the three regions (Masvingo and Matabeleland North) where fertility decline stalled. In addition, adolescent childbearing increased significantly in these two regions, despite a marginal decrease nationally.

The foregoing outcomes underscore the point that a significant predictor of fertility in one country or region of a country may not necessarily be relevant in another country or regions within the same country. Following from the above, we identified three behavioural variables: increases in adolescent fertility, decreases in contraceptive use, and changing proportions of women in union as the most consistent variables associated with stall in fertility at the regional level in Eastern Africa. Also important are factors associated with family planning service environment (decrease in the role of public facilities as sources of family planning services, decrease in approval of family planning, increased unmet need for family planning and unwanted fertility, and declining provision of family planning information on radio). Changes in these variables vary not only across countries but also across regions within the same country and are generally related to observed trends in fertility patterns.

### (f) Common factors associated with stalling fertility decline in Eastern African countries

To identify the common predictors of stall in fertility decline across all regions in the four countries, we further interrogated the institutional, socio-economic and reproductive behaviour variables that indicated association with stall in fertility decline using the probit regression model as explained previously in §4. All variables belonging to each of the three theoretical models are included in the same model to identify the best predictors among the selected independent variables. We analysed the institutional, socio-economic and reproductive behaviour determinants of stall in fertility decline at the regional level. Our outcome variable is whether or not a region experienced a stall in its fertility decline between the third and fourth national demographic and health surveys in each country.

#### (i) Analysis of stall

*Bivariate models *(*table 8, column 1*). Four variables have significant effect on the likelihood of stalled fertility decline at the regional level in the four Eastern African countries under study: change in proportion of women who approve of family planning, change in adolescent fertility, change in ideal number of children, and change in proportion of women in union.

**Table 5.** Change in family planning service environment across regions and fertility trends. n.a., not applicable. **p < 0.05.

region/country	change in					fertility trends
	unwanted fertility	used public service	family planning approval	unmet needs	heard family planning from radio	
*Kenya*						
Nairobi	0.29	−0.25**	0	0.24	n.a.	stall
Central	−0.61**	−0.13**	−0.03**	0.03	n.a.	declining
Coastal	0	0.14	0.01	−0.18**	n.a.	declining
Eastern	−0.07	0.02	−0.11**	−0.07	n.a.	stall
Nyanza	0.33	−0.14**	−0.07**	0.27	n.a.	stall
Rift Valley	0.06	−0.09**	−0.12**	0.03	n.a.	stall
Western	0.33	−0.04	−0.10**	−0.01	n.a.	stall
total	0.08**	−0.08**	−0.10**	0.02	n.a.	stall
*Tanzania*						
Coastal	−0.22**	0.04	0.07**	−0.32**	0.03**	declining
Northern Highlands	0.41	0.01	0.16**	−0.30**	0.04**	declining
Lake	−0.44**	0.04	0.21**	0.19**	0.10**	declining
Central	0.61	0.28	0.01	0.43**	0.04**	stall
Southern Highlands	0.59	0	−0.02	−0.24**	0.02	stall
South	0.41	−0.11**	0.16**	0.25	0.05**	declining
total	0	0.02	0.11**	0	0.04	stall
*Uganda*						
Central	−0.07**	−0.08	n.a.	0.06	−0.02**	declining
Eastern	−0.04	0.07	n.a.	−0.01	−0.04**	increasing
Northern	0.05	0.68	n.a.	0.40**	0.02	declining
Western	0.11	−0.26	n.a.	0.21**	−0.01	declining
total	0	0.06	n.a.	0.13**	−0.03**	declining
*Zimbabwe*						
Manicaland	−0.21	−0.11**	n.a.	0.09	−0.05**	declining
Mashonaland Central	−0.21	−0.08**	n.a.	−0.13	−0.10**	declining
Mashonaland East	−0.11	−0.03**	n.a.	−0.14	−0.10**	declining
Mashonaland West	−0.25	−0.11**	n.a.	−0.04	−0.11**	declining
Matabeleland North	0	0.06**	n.a.	0.18**	−0.10**	stall
Matabeleland South	0	−0.02	n.a.	−0.03	−0.11**	declining
Midlands	0	−0.10**	n.a.	−0.21**	−0.07**	stall
Masvingo	0.11	0.02	n.a.	0.07	−0.10**	stall
Harare/ Chitungwiza	−0.18**	−0.20**	n.a.	0.06	−0.06**	declining
Bulawayo	0.14	−0.24**	n.a.	0	−0.03**	declining
total	−0.11**	−0.08**	n.a.	0	−0.07**	declining

**Table 6.** Changes in socio-economic factors at the regional level and regional fertility trends. **p < 0.05.

region/country	change in				fertility trends
	living in urban area	secondary education	professional work	living in poor households	
*Kenya*					
Nairobi	0	0.04	−0.13	−0.83**	stall
Central	0.37**	0.20**	0.06	−0.68**	declining
Coastal	0.08	−0.06	−0.33**	−0.01	declining
Eastern	−0.21**	−0.24**	−0.08	−0.38**	stall
Nyanza	−0.21**	0.03	−0.25	−0.38**	stall
Rift Valley	0.34**	0.02	−0.34**	−0.11**	stall
Western	−0.05	−0.23**	−0.51**	−0.27**	stall
total	0.08**	0	−0.19**	−0.27**	stall
*Tanzania*					
Coastal	0.17**	0.40**	0.25	0.12**	declining
Northern Highlands	−0.38**	1.29**	0.13	−0.44**	declining
Lake	0.28**	0.49	0.58	−0.08	declining
Central	0.24	0.24	0.87	−0.19**	stall
Southern Highlands	−0.15	−0.06	−1.07	−0.18**	stall
South	−0.14	0.49	0.45	−0.22**	declining
total	0.30**	0.48**	0.12	−0.16**	stall
*Uganda*					
Central	0.01	0.19**	0.28	−0.18**	declining
Eastern	−0.20**	0.20**	0.31	−0.11**	increasing
Northern	0.45**	0.11	0.27	0.04	declining
Western	0.51**	0.16	0.25	−0.20**	declining
total	0.01	0.12**	0.23	−0.08**	declining
*Zimbabwe*					
Manicaland	0.42**	0.21**	0.16	−0.21**	declining
Mashonaland Central	−0.47**	0.15**	0.31	0.15	declining
Mashonaland East	0.33**	0.20**	−0.06	−0.12	declining
Mashonaland West	0.25**	0.24**	0.13	0.04	declining
Matabeleland North	−0.37**	0.05	−0.33**	0.23**	stall
Matabeleland South	0.23**	0.11	−0.03	−0.35**	declining
Midlands	0.07	0.11	0.12	0.06	stall
Masvingo	−0.26**	0.01	−0.66**	0.04	stall
Harare/Chitungwiza	0	0.07**	−0.09	0.13	declining
Bulawayo	0	0.09**	−0.14	0	declining
total	0.01	0.11**	−0.07**	0.03	declining

**Table 7.** Changes in reproductive behaviour factors at regional level and regional fertility trends. **p < 0.05.

region/country	change in				fertility trends
	ideal number of children	contraceptive use	adolescent fertility	women in union	
*Kenya*					
Nairobi	0.10	−0.05	0.50**	−0.06	stall
Central	0	0.06	0.04	−0.11**	declining
Coastal	0.02	−0.05	−0.07	0.02	declining
Eastern	0.21	−0.08	−0.02	−0.01	stall
Nyanza	0	−0.17**	0.10	0.02	stall
Rift Valley	−0.02	−0.07	0.10	−0.01	stall
Western	−0.02	0.22**	0	−0.06	stall
total	0.03	0	0.07	−0.02**	stall
*Tanzania*					
Coastal	−0.08	0.23**	−0.56**	0.01	declining
Northern Highlands	−0.13	0.06	0.13	0.06	declining
Lake	−0.04	0.29**	0.10	0.01	declining
Central	−0.04	0.62**	0.24	0.04	stall
Southern Highlands	−0.02	−0.04	0.36**	0.03	stall
South	−0.08	0.52**	−0.14	−0.09**	declining
total	−0.06	0.17**	−0.01	0.02	stall
*Uganda*					
Central	0.02	−0.03	−0.33**	−0.08**	declining
Eastern	0.07	0.33**	−0.28**	−0.08**	increasing
Northern	−0.06	−0.48**	−0.21**	−0.03**	declining
Western	0	0.12	−0.06	−0.06**	declining
total	0.03	−0.01	−0.24**	−0.06**	declining
*Zimbabwe*					
Manicaland	−0.07	0.26**	0.19**	−0.06**	declining
Mashonaland Central	−0.06	0.11**	−0.17**	0.01	declining
Mashonaland East	−0.06	0.08**	−0.10	−0.05**	declining
Mashonaland West	−0.03	0.06	−0.06	−0.04	declining
Matabeleland North	0	−0.02	0.32**	0	stall
Matabeleland South	−0.02	0.10	−0.44**	−0.07**	declining
Midlands	−0.03	0.18	−0.11	0.01	stall
Masvingo	0.03	−0.02	0.29**	0.03	stall
Harare/Chitungwiza	0	0.07**	−0.05	−0.12**	declining
Bulawayo	−0.02	0.06**	−0.59**	−0.23**	declining
total	−0.02	0.09**	−0.02	−0.04**	declining

**Table 8.** Bivariate and multivariate probit regression models of change in institutional, socio-economic and reproductive behaviour factors on stalled fertility decline at the regional level in Eastern Africa. *p < 0.10; **p < 0.05; ***p < 0.001.

	crude effect	family planning service environment	socio-economic	reproductive	overall
change in unwanted fertility	1.34	1.85			
change in use of public service	−0.07	2.47			
change in proportion approving family planning	−11.35***	−14.81***			−12.71***
change in unmet need for family planning	0.86	3.50			
change in proportion of hearing family planning information on radio	0.25				
change in proportion of women living in urban areas	−1.16		−1.01		
change in proportion of women with secondary+ education	−3.64		−1.91		
change in proportion of women in professional work	−1.70		−0.95		
change in proportion of women living in a poor household	−1.04		−1.48		
change in ideal number of children	8.37**			16.09**	1.89
change in contraceptive use	−0.60			0.58	
change in adolescent fertility	5.53***			5.39***	7.64**
change in proportion of women in union	14.42***			15.12	0.18
_const		−0.43	−0.15	0.23	−11.17
log pseudolikelihood		−4.30	−15.46	−9.43	−4
Wald chi-square		9.62**	9.26*	17.14***	9.97**
*R*^2^		0.57	0.25	0.54	0.60

The likelihood of stalled fertility increases if ideal number of children, adolescent childbearing and proportion of women in union increase. The same trend is observed with respect to increase in unwanted fertility and unmet need but the results are not statistically significant. By contrast, the likelihood of stall is decreased if the proportion of women who approve of family planning increases. Likewise, positive change in proportion of women living in urban areas, women with secondary or higher education, and women in professional employment, as well as increases in contraceptive use are all associated with decrease in the likelihood of stalled fertility decline. However, although these results are theoretically and empirically intuitive, they are not statistically significant.

*Multivariate models*. Our multivariate models include family planning service environment, socioeconomic and reproductive behaviour variables separately and jointly. The results are presented in subsequent columns of table 8. Controlling for other variables, change in approval of family planning is the key family planning service environment factor associated with stall in fertility decline (column 2). Considering the reproductive behaviour model in column 4, change in ideal number of children, change in adolescent fertility and change in proportion of women in union are the key factors driving fertility change in the Eastern African region. In the model combining all the variables (last column), change in the proportion of women who approve family planning and change in adolescent fertility are the most consistent determinants of stall in fertility decline across all four countries and across all regions within them.

### (g) Implications of the stall for future population dynamics in the region

Researchers have suggested that the future of fertility transition in SSA will depend on the future of key socio-economic indicators, including, among others, changes in women's education. Accordingly, if improvements in these socio-economic and family planning service environment indicators that have been realized in the past do not continue throughout the region, then the stalling phenomenon may well spread to more countries. Conversely, stronger progress in improving these indicators in the future would be expected to enhance the likelihood of a second wave of fertility decline in the countries currently experiencing a stall (Shapiro & Gebreselassie 2008).

**Figure 2.**
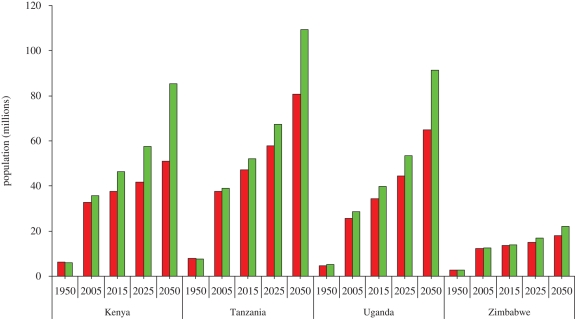
Changes in future population projections before and after the stall in fertility decline (in millions). Red bars, 1998 projections; green bars, 2008 revision).

Despite the acute awareness of the uncertainty intrinsic in population projections (Casterline 2001*a*,*b*), we present and consider the range of demographic futures of the region following the United Nations population projections before and after the incidence of stall was identified in some sub-Saharan countries. Figure 2 summarizes the 1998 population projections for Kenya, Tanzania, Uganda and Zimbabwe before the stall was evident and the recent (2008) revisions after the experience of stall in fertility decline. Apart from Zimbabwe with a continuing decrease in national and regional TFR and where there has been increased out-migration owing to political problems in the country, the 2008 revisions show substantial increases in the population of Eastern African countries; modestly by 2015 but with much more significant growth between 2015 and 2050. These estimates assume the medium variant where TFR is expected to decline to about 2.5 by 2050 in Kenya, Uganda and Tanzania and to 2.1 in Zimbabwe by 2050. For Kenya, if the current level of stall reverses such that fertility levels decline to 4.5 by 2010 and 4.0 by 2015, the population is projected to grow to 85 million by 2050 instead of the 51 million projected by the 1998 revisions. This represents an additional 67 per cent growth burden or an absolute increase of 34 million persons—which is about the current size of the country. Tanzania is now projected to reach 110 million people by 2050, almost tripling its current size. For Uganda the projected growth was revised upwards from 65 million by 2050 to 91 million, an added growth burden of 41 per cent or an absolute increase of 26 million people and a near tripling of its current size. The new projections for Zimbabwe had very marginal increase over the previous projections with only a 2 per cent increase.

Casterline (2001*a*,*b*) had suggested that where mortality declines more rapidly and where economies change more rapidly, the pace of fertility decline will be more rapid. However, controversy regarding the direction of causality between changes in socio-economic conditions at the micro or macro level and changes in fertility remain. At current levels of population growth, reversing the stall in fertility decline in the region is a major development priority if the region is to make progress in achieving national aspirations for food security, universal primary education, increased access to health services, increased employment opportunities and overall economic development goals, including meeting the millennium development goals (MDGs). Achieving such reversals in stalled fertility decline, however, will require specific actions to reverse the trends of increasing levels of desired family sizes, growing negative attitudes towards family planning as well as meeting current high levels of unmet need for family planning and high unwanted childbearing in the region.

## 6. Discussion and conclusions

Our findings are generally consistent with previous studies and raise a number of programmatic and policy relevant questions. In most of the countries, contraceptive prevalence stalled or increasing trends were reversed. Even in places where prevalence has not declined, the percentage increase decelerated from one survey period to the next over the last decade. In Kenya, Westoff (2006) observed an increase in contraceptive discontinuation rate from 28 per cent in 1998 to 33 per cent in 2003, which is linked to side effects associated to hormonal methods particularly pills and injectables. The rise in adolescent fertility is also found to be associated with stall in contraceptive use among Kenyan youths. The implications of the weakened family planning service environment for adolescent childbearing and fertility outcomes among other marginalized groups require specific attention. Not only are the fertility preferences of adolescents changing in favour of larger family sizes, but also their attitude towards family planning is growing increasingly more negative. In Kenya, for instance, the proportion of adolescents aged 15–19 years who disapproved of family planning increased by 66 per cent between 1998 and 2003 from 13.4 per cent to 22.4 per cent. Reaching this population group, who will be the drivers of future fertility patterns, is crucially important.

The observed increase in desire for large families evokes an important question. Ezeh & Dodoo (2001) suggested that religious systems that equate pronatalism as divine blessing and infertility as a curse could motivate reversals in fertility preferences. Westoff & Cross (2006) found a shift towards large fertility preferences among Muslims in Kenya. The rise in certain Pentecostal movements, especially among young people and the link with doctrines opposed to modern contraceptive use deserves further investigation (Gregson *et al*. 1999; Agadjanian 2001; Agha *et al*. 2006).

We found stall in fertility decline is positively correlated with the proportion of women with no education. This outcome is congruent with the conclusion that positive shifts in educational attainment contributed substantially to the decline in fertility in Kenya (Ezeh & Dodoo 2001). This finding underscores the perspective that improving access to female education is imperative to continued improvement in contraceptive use and sustained fertility transition in the region. In this light, national policies based on human development (education and health) have been identified as a key factor for achieving accelerated fertility decline in the region (Emina 2002). Although the socio-economic variables are not significant in the multivariate analysis, the finding that regions with large reversals in fertility decline were more likely to experience reductions in the proportion of women with secondary or higher education or the proportion working in formal employments, suggest the importance of these socio-economic factors in defining future fertility trends in the region.

In conclusion, the stall in fertility in Kenya and Tanzania, and the marginal fertility decline in Uganda at pre-transition levels sums the view of a truly dramatic population growth in these countries over the next 20 years. Given the strong linkages between rapid population growth and poor economic growth and development, it is evident that many countries in SSA will not meet the MDG targets. Initiating a second wave of fertility transition in the region, however, will require a refocusing of attention to family planning programmes as a critical part of development agenda in the region.

## 7. Opportunities for addressing stalled fertility in the region

Our analysis identifies some potential opportunities for addressing stalled fertility in the region. First, there is the potential to look beyond national level fertility indicators, with particular attention on complex contextual nuances and regional differentials within different countries and across subgroups. Second, is the need to reinvigorate campaigns on contraceptive use and engaging policy makers with evidence to increase budgetary allocation towards increasing contraceptive prevalence. It is important that a new MDG target on universal access to reproductive health services has been introduced. Given the lag in the introduction of this target, conscientious efforts are needed to reverse the impact of the many years of neglect of family planning programmes in the region. Efforts to achieving universal access to reproductive health services must emphasize the rebuilding of systems of family planning service delivery that crumbled under years of neglect. The public sector must be strengthened to respond effectively to addressing the high levels of unwanted childbearing and unmet need for family planning.

Finally, the finding of increased adolescent childbearing is consistent with the prognoses that Africa's adolescent reproductive health yielded the worst indicators relative to other regions of the world and advances further evidence that the onset of sexual encounters in the region happens in the context of low levels of contraception and protective measures (Okonofua 2007). Following the consequent high burden of related debilitating outcomes including sexually transmitted infections, HIV/AIDS, unwanted pregnancies, unsafe abortion, and high fertility rates among youths, the challenge of interventions dealing with the consequences of adolescent sexuality remains enormous in the region. The strong relationship between adolescent childbearing and stall in fertility decline across all countries, speaks to the challenge of addressing issues relating to adolescent access to contraceptive services in the region. The hindering roles of parents, relatives, religious groups and even service providers in limiting adolescent access to sexual and reproductive health information and services even when they are available have previously been identified (Adedimeji 2005; Mberu 2008). The challenge for policy and programmes will therefore be how to reconcile parental and societal interest in adolescent sexual behaviour with the need to create opportunities for access to protective services for youths. Ability of the region to achieve another wave of fertility decline may hinge heavily on the region's ability to effectively reach and meet adolescents' sexual and reproductive health needs.

## Endnotes

1 Stall is defined operationally as a failure of the national TFR to decline between two (most recent) DHS surveys after an established trend of decline in national fertility (Bongaarts 2006).

2 The four countries chosen are those with four rounds of nationally representative surveys to allow for monitoring of fertility trends and observing clear changes over time.

3 Report of the Secretary-General. United Nations Economic and Social Council, New York: commission on population and development, 37th session, 22–26 March 2004).

4 Rather than using wealth index, we define poor households as those living in dwelling units without piped water, flush toilet, electricity, TV and fridge in urban areas, and those living in houses built with non-durable roof and floor materials and without bicycle and radio in rural areas.
